# Impact of Evidence‐Based Dentistry Education on Dental Students' Critical Appraisal Skills: A Prospective Cohort Study

**DOI:** 10.1111/eje.70066

**Published:** 2025-10-28

**Authors:** Carlota Inês Duarte de Mendonça, António Duarte Sola Pereira da Mata, Bruno Filipe da Costa Rosa, Joana Rita Oliveira Faria Marques, João Miguel Lourenço Silveira, Duarte Nuno da Silva Marques

**Affiliations:** ^1^ Biology and Oral Biochemistry Group, Faculty of Dental Medicine University of Lisbon Lisbon Portugal; ^2^ Center for Evidence‐Based Dental Medicine, Faculty of Dental Medicine University of Lisbon Lisbon Portugal; ^3^ Faculty of Dental Medicine University of Lisbon Lisbon Portugal; ^4^ Hugo Madeira Clinic—Advanced Aesthetics and Implantolo Lisbon Portugal; ^5^ LIBPhys‐FCT UIDB/04559/2020, Faculty of Dental Medicine University of Lisbon Lisbon Portugal; ^6^ Institute of Implantology Lisbon Portugal

**Keywords:** dental education, evidence‐based dentistry, health impact assessment

## Abstract

**Objective:**

This study aimed to assess the impact of evidence‐based dentistry (EBD) education on the critical appraisal abilities of dental students.

**Methods:**

A prospective cohort study was conducted with students from the Faculty of Dental Medicine in Lisbon. After Local Ethics Committee approval and participants' informed consent, they completed the Cochrane Collaboration's Tool Assessment Risk of Bias 5.1.0 for two pre‐selected articles, previously appraised by experts (reference answers). Questionnaires were administered at two time points, T0 (baseline) and T1 (post‐training), with either 4.5 or 16 h of EBD instruction in between. Intra‐student and inter‐operator agreement were assessed using the Fleiss multirater kappa coefficient. Changes in the proportion of correct responses between T0 and T1 were evaluated using the McNemar–Bowker test, with significance set at *p* < 0.05.

**Results:**

Recruitment began in January 2022, and 81 students enrolled (13.6% male, 86.4% female; mean age 26.84 ± 6.51 years). Response rates were 100% at T0, and 97.53% (paper A) and 98.77% (paper B) at T1. The Fleiss multirater kappa coefficient indicated slight agreement between T0 and T1 responses, with increased inter‐operator agreement following training. A statistically significant improvement in critical appraisal performance was observed post‐training (McNemar–Bowker Test = 395.450, df = 6, *p* = 0.001). Subgroup analysis based on training duration and students' self‐reported prior EBD education revealed no significant differences in outcomes.

**Conclusions:**

EBD education significantly improves the critical appraisal abilities of dental students, emphasising the positive impact of EBD training.

## 1 Introduction

The American Dental Association defines evidence‐based dentistry (EBD) as a patient‐centred approach that integrates systematic assessments of clinically relevant scientific evidence with a dentist's expertise and the patient's needs and preferences [1, 2]. Implementing EBD as the gold standard in clinical practice requires specific skills that develop over time [1, 3]. However, many clinical decisions still rely on experiential knowledge rather than evidence, highlighting a persistent gap between research and practice [4]. This issue is central to the concept of ‘knowledge translation’, which seeks to transform research findings into practical applications that enhance healthcare outcomes.

Despite its importance, advancing EBD faces multiple challenges, including the vast volume of research, variability in evidence quality, conflicting findings, scientific illiteracy and an imbalance between knowledge production and effective dissemination [5, 6]. These barriers underscore the necessity of foundational EBD training to optimise the use of evidence‐based tools [1, 5]. As a result, incorporating EBD into dental curricula has become increasingly urgent to ensure future professionals can critically appraise and apply scientific evidence in practice [7, 8].

While many training programs aim to equip students with EBD methodology, limited research assesses whether students effectively integrate EBD principles into clinical decision‐making. Taylor et al. [9] conducted a controlled trial evaluating a critical appraisal skills workshop, but the intervention did not significantly improve students' confidence or attitudes toward evidence. Similarly, Madhavji et al. [10] and Khami et al. [11] reported that American orthodontists and Iranian dental students demonstrated low EBD knowledge despite expressing positive attitudes. In contrast, Najafi and Asgari [12] found that EBD training positively impacted senior students, and Garg et al. [13] suggested that critical appraisal training enhances clinical confidence.

Although many universities include EBD in their curricula, the structure and teaching methods vary widely, and their effectiveness remains unvalidated. This inconsistency highlights the need for robust evaluation strategies to assess whether EBD education translates into improved clinical practice. Ensuring that future dental professionals acquire EBD skills is essential for fostering evidence‐based decision‐making, particularly in the critical appraisal of scientific literature to select high‐quality evidence for patient care.

The primary objective of this prospective cohort study was to assess the impact of EBD education on the critical appraisal skills of degree‐seeking students (undergraduate and doctoral) as well as non‐degree students (postgraduate). We hypothesised that there would be differences in agreement and consistency in responses to the bias assessment instrument between different measurement times, comparing student responses to those of experts.

## 2 Materials and Methods

This prospective cohort study adhered to the STROBE Statement [14] and was approved by the Local Ethical Committee of the Faculty of Dental Medicine, University of Lisbon. Conducted at the same institution from 2021 onward, it required students' implied consent before enrolment. Institutional Review Board authorisation was obtained prior to initiation (code CE‐FMDUL20255).

### 2.1 Sample and Setting

Participants were recruited from undergraduate, PhD and postgraduate programs at the Faculty of Dental Medicine. A total of 175 students were enrolled to account for a 20% dropout rate, ensuring a final sample of 140, with a statistical power of 0.81 for detecting a discordant proportion sum of 0.1 [15, 16]. The study used a convenience sample from a single university, where enrolment limitations led to each student representing *n* = 2.

### 2.2 Procedure/Assessment

Two randomised controlled trials (RCTs) were critically appraised at two time points: T0 (before EBD education) and T1 (after EBD education).

The EBD program consisted of weekly in‐person theoretical lessons taught by a faculty member, each lasting 1 h. Undergraduate students received 4.5 h of training, while PhD and postgraduate students completed 16 h.

Undergraduate topics included: (1) introduction to EBD, (2) steps of clinical investigation, (3) scientific project design, (4) data collection and presentation, and (5) integration of research and clinical practice. The advanced curriculum covered: (1) EBD methodology, (2) development of evidence‐based clinical guidelines, (3) PICO criteria, (4) CASP checklists for RCTs and systematic reviews, (5) validity, relevance and statistical analysis, (6) literature appraisal, and (7) practical exercises using CASP tools.

Undergraduate students were evaluated through the development of a protocol for a scientific project, whereas PhD and postgraduate students were assessed using the risk of bias tool questionnaire at T1.

The survey, supervised by an external observer, collected demographic data (ID, gender, birth date, enrolment, graduation year and EBD training hours). The appraisal questionnaire was adapted from the Cochrane Collaboration's risk of bias tool (version 5.1.0) [17]. To prevent bias, all questionnaires were completed individually and in person.

### 2.3 Variables

Primary outcomes included the number of correct responses at baseline (T0) and post‐intervention (T1), along with intra‐student agreement on the bias assessment tool. Secondary outcomes involved a qualitative analysis of response types in relation to the educational content. Total and prior EBD training hours were considered confounding variables.

### 2.4 Statistical Analysis

Data analysis was conducted using IBM SPSS (version 29.0.1.1). Intra‐student agreement and agreement with expert bias assessments were evaluated using Fleiss multirater kappa [18] with a 95% confidence interval. The McNemar–Bowker Test compared correct response proportions between T0 and T1, with missing values assigned to the most frequent category. Expert evaluations followed Cochrane team guidelines, referencing systematic reviews CD003067 [19] and CD001168 [20]. Statistical significance was set at *p* < 0.05. Justifications for risk of bias were categorised as ‘Not acquired’, ‘Acquired but not applied’, ‘Acquired and applied’ or ‘No reply’.

## 3 Results

### 3.1 Descriptive Data

**FIGURE 1:**
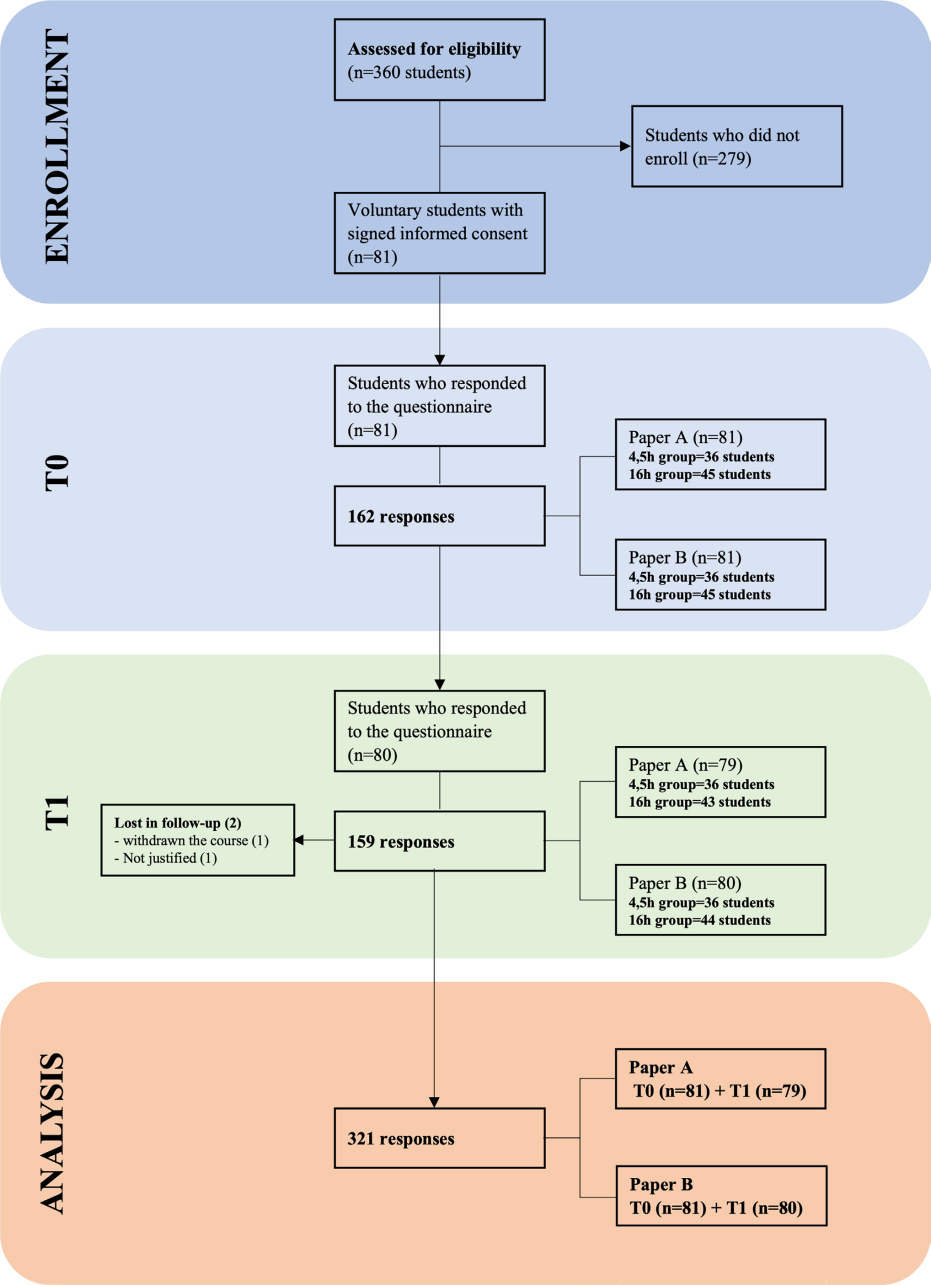
Report number of individuals at each stage of the study.

The flow diagram of the study participants is illustrated in Figure 1. The study included 81 students (13.6% male, 86.4% female) with an average age of 26.84 ± 6.51 years (range: 20–47). Forty‐seven students were in degree‐conferring programs (36 undergraduates, 11 PhD), while 34 were in postgraduate programs. Participants were divided based on EBD education exposure for subgroup analysis: 36 students (undergraduates) received 4.5 h and 45 (PhD and postgraduate) received 16 h. One student withdrew before completing T1 and another only partially completed the questionnaire. Despite these losses, the final sample of 79 students maintained sufficient statistical power.

### 3.2 Main Results

#### 3.2.1 Primary Outcomes

##### 3.2.1.1 Intra‐Student and Inter‐Operator (Student vs. Expert) Agreement

Intra‐student agreement between T0 and T1 (Table 1) responses was low, with a kappa coefficient of 0.086 (95% CI [0.051; 0.121], *p* = 0.001). Most domains showed no significant agreement, except for ‘Allocation Concealment’ (kappa = 0.114, 95% CI [0.017; 0.211], *p* = 0.022). At T0, student‐expert agreement was poor (−0.057, 95% CI [−0.094; −0.019], *p* = 0.003) (Table 2), but improved significantly at T1 (kappa = 0.274, 95% CI [0.226; 0.321], *p* = 0.001) (Table 3). While student‐expert agreement remained low across most domains, ‘Attrition Bias’ showed improvement (kappa = 0.171, 95% CI [0.037; 0.305], *p* = 0.013), whereas ‘Random Sequence Generation’ and ‘Performance Bias’ retained non‐significant agreement levels.

**TABLE 1:** Fleiss multirater kappa and *p*‐values for intra‐student agreement individually for each domain of The Cochrane Collaboration's Tool for assessing risk of bias 5.1.0.

Intra‐student agreement (student × T0 vs. T1)				
			Kappa [95% CI]	*p*
T0 vs. T1 (*n* = 80 students; 159 effective answers)	Domain: Selection bias	Random sequence generation	−0.093 [−0.195; 0.010]	0.076
		Allocation concealment	0.114 [0.017; 0.211]	0.022*
	Domain: Performance bias	Blinding of participants and personnel	−0.046 [−0.143–0.050]	0.347
	Domain: Detection bias	Blinding of outcome assessment	−0.043 [−0.137; 0.050]	0.363
	Domain: Attrition bias	Incomplete outcome data	−0.071 [−0.166; −0.025]	0.147
	Domain: Reporting bias	Selective reporting	0.025 [−0.066; 0.116]	0.589
	Other bias	Other sources of bias	−0.040 [−0.136; −0.057]	0.419

* Statistically significant result.

**TABLE 2:** Fleiss multirater kappa and *p*‐values for inter‐operator agreement (T0) individually for each domain of The Cochrane Collaboration's Tool for assessing risk of bias 5.1.0.

Inter‐operator agreement (T0; student vs. expert)				
			Kappa [95% CI]	*p*
T0 (*n* = 81 students; 162 effective answers)	Domain: Selection bias	Random sequence generation	−0.213 [−0.318; −0.108]	0.001*
		Allocation concealment	−0.261 [−0.369; −0.152]	0.001*
	Domain: Performance bias	Blinding of participants and personnel	−0.304 [−0.404; −0.203]	0.001*
	Domain: Detection bias	Blinding of outcome assessment	−0.376 [−0.479; −0.273]	0.001*
	Domain: Attrition bias	Incomplete outcome data	−0.043 [−0.141; 0.054]	0.383
	Domain: Reporting bias	Selective reporting	−0.417 [−0.524; −0.310]	0.001*
	Other bias	Other sources of bias	−0.455 [−0.558; −0.352]	0.001*

* Statistically significant result.

**TABLE 3:** Fleiss multirater kappa and *p*‐values for inter‐operator agreement (T1) individually for each domain of The Cochrane Collaboration's Tool for assessing risk of bias 5.1.0.

Inter‐operator agreement (T1; student vs. expert)				
			Kappa [95% CI]	*p*
T1 (*n* = 80 students; 159 effective answers)	Domain: Selection bias	Random sequence generation	−0.038 [−0.164; 0.088]	0.554
		Allocation concealment	−0.141 [−0.259; −0.023]	0.019*
	Domain: Performance bias	Blinding of participants and personnel	−0.093 [−0.214; 0.028]	0.132
	Domain: Detection bias	Blinding of outcome assessment	−0.139 [−0.255; −0.023]	0.019*
	Domain: Attrition bias	Incomplete outcome data	0.171 [0.037; 0.305]	0.013*
	Domain: Reporting bias	Selective reporting	−0.294 [−0.408; −0.181]	0.001*
	Other bias	Other sources of bias	−0.496 [−0.605; −0.387]	0.001*

* Statistically significant result.

#### 3.2.2 Proportion of Correct Answers

The percentage of correct answers increased markedly from 325 (29.2%) at T0 to 650 (57.3%) at T1, nearly doubling after EBD training. Additionally, the number of unanswered questions decreased significantly from 416 at T0 to just 35 at T1. A significant association was found between students' critical appraisal performance before and after EBD training (McNemar–Bowker Test = 395.450, df = 6, *p* = 0.001) (Table 4).

**TABLE 4:** Proportion of correct answers between T0 and T1 (student vs. experts) [McNemar–Bowker Test column]; *p* value corresponds to significance. Number of responses correct before (T0) and after (T1), ce—based Dentistry.

		Total sample of students (*N* = 79)			T0				T1			
		McNemar–Bowker Test	df	*p*	Correct	Incorrect	Unclear	No reply	Correct	Incorrect	Unclear	No reply
Domain: Selection bias	Random sequence generation	60.098	6	0.001*	79	26	16	38	144	5	10	0
	Allocation concealment	54.822	6	0.001*	68	14	25	52	106	27	25	1
Domain: Performance bias	Blinding of participants and personnel	68.705	6	0.001*	50	33	29	47	123	16	20	0
Domain: Detection bias	Blinding of outcome assessment	76.781	6	0.001*	35	41	21	62	106	18	31	4
Domain: Attrition bias	Incomplete outcome data	61.260	6	0.001*	44	28	30	57	91	51	15	2
Domain: Reporting bias	Selective reporting	73.874	6	0.001*	30	24	25	80	63	58	30	8
Other bias	Other sources of bias	61.088	6	0.001*	19	33	27	80	17	92	30	20
	Total	395.450	6	0.001*	325	199	173	416	650	267	161	35

*Note:* ‘Correct answer’ was defined as when both student and expert answered ‘Low risk of bias’ or ‘High risk of bias’. ‘Incorrect answer’ was when the student's answer differed from the experts. ‘Unable to identify’ corresponded to ‘Unclear risk of bias’.

* Statistically significant result.

#### 3.2.3 Subgroup Analyses

Subgroup analysis comparing students with 4.5 and 16 h of EBD training found no significant differences in correct answers, though both groups showed significant improvements in critical assessment skills. Intra‐student agreement remained low across groups, with a slight, non‐significant improvement in ‘Performance Bias’ for the 4.5‐h group and a decrease in ‘Reporting Bias’ for the 16‐h group.

For inter‐operator agreement, the 4.5‐h group showed a slight but non‐significant improvement in ‘Attrition Bias’ at T0. At T1, agreement in this domain was poor for the 4.5‐h group but reached moderate significance for the 16‐h group (kappa = 0.413, 95% CI [0.220; 0.305], *p* = 0.001).

No significant differences in correct answers were found between training durations, but both groups improved post‐training (Table 5).

**TABLE 5:** Proportion of correct answers between T0 and T1 (student vs. experts) according to subgroup analysis [McNemar–Bowker Test column]; *p* value corresponds to significance.

		4.5 h of EBD education (*N* = 36)			16 h of EBD education (*N* = 90)		
		McNemar–Bowker Test	df	*p*	McNemar–Bowker Test	df	*p*
Domain: Selection bias	Random sequence generation	16.825	6	0.010*	47.000	3	0.001*
	Allocation concealment	27.067	6	0.001*	48.778	6	0.001*
Domain: Performance bias	Blinding of participants and personnel	17.865	5	0.003*	24.505	6	0.001*
Domain: Detection bias	Blinding of outcome assessment	32.471	6	0.001*	47.900	6	0.001*
Domain: Attrition bias	Incomplete outcome data	13.048	6	0.042*	24.505	6	0.001*
Domain: Reporting bias	Selective reporting	26.014	6	0.001*	53.697	6	0.001*
Other bias	Other sources of bias	24.505	6	0.001*	44.840	6	0.001*
	Total	99.609	6	0.001*	298.526	6	0.001*

* Statistically significant result.

Prior EBD education did not significantly impact critical appraisal skills. At T0, student‐expert agreement was poor (−0.144, 95% CI [−0.253; −0.036], *p* = 0.009), reflecting initial difficulties. At T1, improvements were modest overall, though ‘Random Sequence Generation’ achieved near‐perfect agreement and ‘Attrition Bias’ showed substantial agreement (kappa = 0.693, 95% CI [0.255; 1.131], *p* = 0.002).

#### 3.2.4 Secondary Outcomes

Integrating EBD education into the curriculum is crucial for developing critical appraisal skills and enhancing evidence‐based clinical decision‐making. At T0, 82.28% of responses (933/1134) were classified as ‘Do not know,’ highlighting students' lack of knowledge with the most challenging domains being ‘Detection Bias’ (12.35%), ‘Reporting Bias’ (12.70%) and ‘Other Bias’ (13.14%).

At T1, after EBD education, 52.65% of responses (586/1113) were categorised as ‘Acquired and Applied,’ indicating correct answers with appropriate justifications. However, 16.26% of responses (181) fell under ‘Acquired but not applied,’ suggesting partial understanding, and 31.09% (346) remained ‘Not Acquired,’ indicating a failure to integrate training.

Despite improvements, students still struggled with ‘Reporting Bias’ (6.92%), ‘Other Bias’ (10.15%) and ‘Attrition Bias’ (6.02%). In contrast, they showed better comprehension in ‘Selection Bias’ (Random Sequence Generation and Allocation Concealment), suggesting greater effectiveness of education in these domains.

## 4 Discussion

This study demonstrates significant positive improvements in the consistency and agreement of students' responses on a bias assessment tool following EBD education, supporting the study hypothesis. The Fleiss multirater kappa coefficient indicated slight agreement between T0 and T1 responses and improved inter‐operator agreement after EBD education, which underscores the positive influence of EBD education on students' critical appraisal abilities. The Fleiss multirater kappa coefficient for intra‐student agreement between T0 and T1 responses remained low, highlighting the need for structured EBD education. The observed shift in student answers between time points suggests a positive progression in comprehension, as students' baseline responses (T0) likely reflected limited understanding of bias assessment concepts.

At baseline (T0), the Fleiss multirater kappa coefficient for inter‐operator agreement was poor, indicating students' initial lack of skill in accurately appraising published evidence. This gap underscores the importance of EBD education in cultivating critical appraisal skills. By T1, the kappa coefficient improved to fair agreement, indicating a measurable enhancement in students' appraisal skills, thus validating the role of EBD education in fostering critical thinking skills. The significant increase in correct answers between T0 and T1, nearly doubling, underscores the essential role of EBD education in the development of students' critical appraisal skills. However, for specific questions, such as those assessing ‘Allocation Concealment,’ ‘Incomplete Outcome Data,’ ‘Selective Reporting,’ and ‘Other Sources of Bias,’ we observed an increase in incorrect answers at T1. This trend suggests that students have also developed the ability to critically assess and problematise. Although they overmastered the question ‘Allocation Concealment,’ in article B, the sentence providing the ‘Low Risk of Bias’ for that domain was unpublished. In the justification field, it is evident that students fully understood the question being asked, but they do not have access to the information required to provide the correct answer.

Subgroup analysis did not reveal significant differences between students with varying EBD exposure, likely due to the comprehensive and uniform approach to EBD instruction across curricula. Additionally, previous EBD training reported by students did not appear to influence performance at the T0 baseline evaluation. However, at T1, inter‐operator agreement showed greater improvement compared to the initial analysis. These findings highlight the need for continuous education, which plays a crucial role in consolidating knowledge. In other words, despite prior training, students do not effectively apply their EBD knowledge in clinical practice.

This study was designed to assess the impact of EBD education on dental students' critical appraisal skills. Previous research in this field has primarily focused on evaluating the awareness, perceptions and attitudes of dental students toward EBD, as well as identifying major barriers to its implementation [21]. However, there is a notable gap in studies specifically assessing the effectiveness of EBD education and its integration into dental curricula. The only study that directly evaluated students' critical appraisal abilities was the one published by Taylor and his team [9], which concluded that a half‐day (3‐h) workshop did not result in statistically significant improvements in critical appraisal skills, such as methodology or generalizability. Our study challenges this conclusion, suggesting that the observed differences may be attributed to the educational program applied during the teaching period. This finding underscores the need to standardise teaching methods for EBD skills.

One of the goals of our team was to develop a pragmatic cohort study, aligned with the current research focus on practice‐based research. We chose a real‐world setting, collected and analysed real‐world data, and aimed to understand the challenges students face in understanding and applying EBD skills in real situations.

Our methodology incorporated a critical appraisal tool to address critical thinking as a core educational outcome, with a longitudinal design to assess the educational impact over time, which offers a robust framework for evaluating higher education outcomes [22]. The Cochrane risk of bias tool was selected for its structured format combining multiple‐choice items with justification fields, reducing the likelihood that correct answers are given by chance and providing deeper insight into students' conceptual understanding. This widely accessible tool also has reference answers published in Cochrane Reviews [23, 24], making it both a standardised and practical choice for evaluating dental students' skills.

The questionnaires at different time measurements (T0 and T1) were administered by the same operator, ensuring individual completion of the tool by the students. Anonymity was preserved throughout the data analysis to further limit reporting bias. Students were contacted at T1 to encourage completion and minimise dropout, as the time commitment required for survey completion may impact participation. A limitation of our study is that students were aware that the articles evaluated in T0 would be the same in T1, potentially prompting them to search for correct answers online and study them for the second evaluation. We considered this limitation a failure in controlling all confounding variables. However, the justification fields in the answers provided by the students differed from the original responses given by the experts, leading us to speculate that they did not search for the answers online. Additionally, the investigators never mentioned that the selected RCTs were part of systematic reviews of the Cochrane Library.

The use of a convenience sample from a single university may compromise the external validity of our findings, as results could differ in other institutions depending on the specific EBD curricula hours and implementation. Future studies should include multiple universities where EBD curricula are already implemented. Furthermore, we propose reviewing and potentially enhancing the EBD curricula to improve the effectiveness of training and strengthen the generalizability of future findings.

Although EBD training has been shown to enhance students' short‐term knowledge acquisition and critical appraisal skills, its long‐term influence on clinical behaviour and patient care remains insufficiently characterised [25, 26]. Existing systematic reviews and longitudinal studies suggest that initial improvements in EBD competencies frequently diminish over time, as new graduates encounter contextual barriers such as time constraints, financial pressures and workplace culture, which may hinder the sustained application of evidence‐based principles in daily practice [27, 28, 29]. Despite some evidence indicating that predoctoral EBD education fosters continued engagement with peer‐reviewed literature, robust data demonstrating enduring behavioural change in clinical decision‐making is limited, largely due to methodological heterogeneity and the predominance of observational study designs [30].

In this context, the primary focus of the present study was to evaluate short‐term improvements in EBD‐related skills following an educational intervention. Future research should extend these findings by investigating the long‐term impact of EBD training on clinical practice and the integration of evidence‐based decision‐making among dental practitioners.

## 5 Conclusions

This study highlights the crucial role of EBD education in enhancing dental students' critical appraisal skills. The findings emphasise the positive impact of EBD training on research evaluation and advocate for its integration into dental curricula. Such integration is essential for bridging the gap between evidence and clinical practice, ensuring future dental professionals make informed, evidence‐based decisions in patient care.

## Conflicts of Interest

The authors declare no conflicts of interest.

## Data Availability

Data available on request from the authors. The data that support the findings of this study are available from the corresponding author upon reasonable request.
